# Case Report: Suspected hereditary spontaneous hydrocephalus in a Beagle pedigree

**DOI:** 10.3389/fvets.2026.1857970

**Published:** 2026-07-03

**Authors:** Jing-Wen Fu, Jia-Yi Liu, Ding-Jun Jin, Jin-Juan Huang, Si Yang, Jia-Kun Deng, Yun Gao, Jiawei Han, Yefen Liu, Yaowen Xing, Yan-Hu Liu, Ya-Ping Zhang

**Affiliations:** 1 State Key Laboratory of Genetic Evolution and Animal Models and Yunnan Key Laboratory of Molecular Biology of Domestic Animals, Kunming Institute of Zoology, Chinese Academy of Sciences, Kunming, China; 2 University of Chinese Academy of Sciences, Beijing, China; 3 Criminal Investigation Police University of China (CIPUC), Shenyang, Liaoning, China; 4 KIZ-CUHK Joint Laboratory of Bioresources and Molecular Research in Common Diseases, Kunming Institute of Zoology, Chinese Academy of Sciences, Kunming, China; 5 United Imaging Healthcare, Shanghai, China; 6 Bio-X Center for Interdisciplinary Innovation, Yunnan University, Kunming, China

**Keywords:** Beagle pedigree, hereditary, MRI, spontaneous, ventriculomegaly

## Abstract

**Introduction:**

Hydrocephalus is one of the most common malformations in dogs and poses considerable treatment challenges in veterinary clinical practice. Ventricular enlargement may represent an early manifestation of hydrocephalus and is commonly quantified by the ventricle-to-brain index (VBI). This report presents a Beagle pedigree with suspected hereditary lateral ventricular enlargement, confirmed by VBI calculated from magnetic resonance imaging (MRI).

**Case description:**

A 1.4-year-old female Beagle presented with severe hydrocephalus (VBI: 0.72) and complete absence of the parietal lobe. Its sire (ID: 11771, VBI = 0.61) showed hydrocephalus, whereas its dam had minimal lateral ventricular enlargement (VBI = 0.52), implying that the severe hydrocephalus in dog 90044 may have been paternally inherited. Additionally, a second cousin of dog 11771 displayed hydrocephalus, reinforcing the suspicion of a hereditary basis for the condition. To further investigate, we collated phenotypic data from 37 surviving offspring (out of 41) spanning six litters sired by dog 11771. To investigate age-related changes in lateral ventricular volume, we divided the pedigree members into three groups: puppies (<6 months old), juveniles (6–12 months old), and adults (≥12 months old). When assessed using a four-tier VBI classification, a Jonckheere–Terpstra test revealed that VBI and severity grades increased significantly with age, demonstrating progressive lateral ventricular enlargement from puppyhood to adulthood. To further investigate whether the VBI of the 11771 pedigree was abnormally enlarged, we compared its VBI and severity grades to those from 24 healthy Beagles (nine juveniles and 15 adults). Age-stratified comparisons showed that the VBI and severity grades of both juveniles and adults in this pedigree were significantly higher than those of controls.

**Conclusion:**

Although limited by a small sample size from a single pedigree and the absence of long-term follow-up, this case report describes a Beagle pedigree with suspected hereditary, age-dependent progressive lateral ventricular enlargement. This finding provides a valuable reference for the early detection of hydrocephalus, evaluation of treatment efficacy, and assessment of prognostic outcomes.

## 1 Introduction

Hydrocephalus is characterized by pathological accumulation of cerebrospinal fluid (CSF) within the ventricular system, resulting in ventricular enlargement and increased intracranial pressure (1). In dogs, hydrocephalus is one of the most common congenital neurological malformations, and its clinical features and imaging manifestations have been studied for over a century (1). The reported incidence of congenital anomalies in neonatal canines is approximately 7%, with hydrocephalus consistently recognized as a major cause among these malformations (2). Recently, a large-scale analysis of 494 canine brain MRI and/or computed tomography (CT) scans with intracranial injuries, reported that hydrocephalus was the most prevalent abnormality, occurring in 34 of 55 dogs (61.81%) with encephalic malformations (3). The etiology of hydrocephalus is multifactorial and heterogeneous, broadly classified into congenital and acquired forms (1, 4, 5). Congenital hydrocephalus is frequently attributed to genetic predispositions and aberrant embryonic development (4, 6). Although clinical signs of congenital hydrocephalus may manifest at birth, the condition is most frequently diagnosed between 2 and 3 months of age and follows a highly heterogeneous clinical course (4, 7). It frequently occurs in small brachycephalic dog breeds such as Chihuahuas, Maltese, Yorkshire Terriers, Toy Poodles and Pekingese (1, 4, 8). This breed predisposition may be attributed to artificial selection for brachycephaly, which results in altered cranial cavity morphology (9). Interestingly, despite being a mesaticephalic breed, Beagles also exhibit a high prevalence of asymptomatic ventriculomegaly (10). Currently, pedigree analysis and the genetic basis of canine hydrocephalus remain largely unknown. In contrast, acquired hydrocephalus commonly arises secondary to pathologies including intracranial neoplasms, inflammation, hemorrhage, or traumatic injury (1, 6). However, both forms can lead to severe neurological dysfunction or even become life-threatening if not diagnosed and treated promptly.

Hydrocephalus can be reliably diagnosed through advanced diagnostic imaging modalities, specifically ultrasonography, CT, and MRI, which facilitate precise visualization of ventricular enlargement and associated pathoanatomical features (4). Among these, ultrasonography serves as a critical diagnostic technique for canine fetal hydrocephalus (11, 12). MRI, particularly at high-field strengths (e.g., 3.0T), enables superior soft tissue contrast resolution and high-resolution thin-slice acquisitions, making it the most effective and commonly used non-invasive method for diagnosing canine hydrocephalus. T2-weighted imaging is highly sensitive to alterations in water proton relaxation times, with CSF appearing characteristically hyperintense. The ventricle-to-brain index (VBI), calculated as the ratio of the greatest continuous span between the internal margins of ventricles to the maximal breadth of the cerebral parenchyma measured on the exact same T2-weighted slice, is commonly used to quantify the degree of ventricular enlargement in dogs (13, 14). A validated MRI-based grading system (grade 0 to grade 5) demonstrated excellent interobserver agreement and a strong correlation with the VBI (14). A VBI threshold of ≥0.5 has been proposed to identify periventricular tissue alterations and predict potential clinical manifestations (15), whereas a VBI threshold of 0.6 is used to differentiate clinically relevant internal hydrocephalus from asymptomatic ventriculomegaly (13).

Treatments for internal hydrocephalus continue to advance with the implantation of a ventriculoperitoneal shunt (VPS) system serving as the cornerstone intervention (4, 7). However, shunts may be complicated by obstruction, infection and over-drainage, necessitating regular monitoring and potential surgical revisions (16, 17). In some cases, medications reducing the production of CSF, either alone or in combination with shunting, are also recommended as alternatives (4). However, several studies have reported either no significant efficacy or only marginal benefits with those agents (18–21).

This case report presents a Beagle pedigree with suspected hereditary lateral ventricular enlargement. A significant disparity in the grade distribution of lateral ventricular enlargement was observed between the affected pedigree cohort and unrelated control Beagles. Given the critical role of animal models in novel treatment development and the status of Beagle as a widely used standard experimental breed, this family holds unique value for advancing both therapeutic innovations and early diagnostic approaches for hydrocephalus.

## 2 Case description

**Figure 1:**
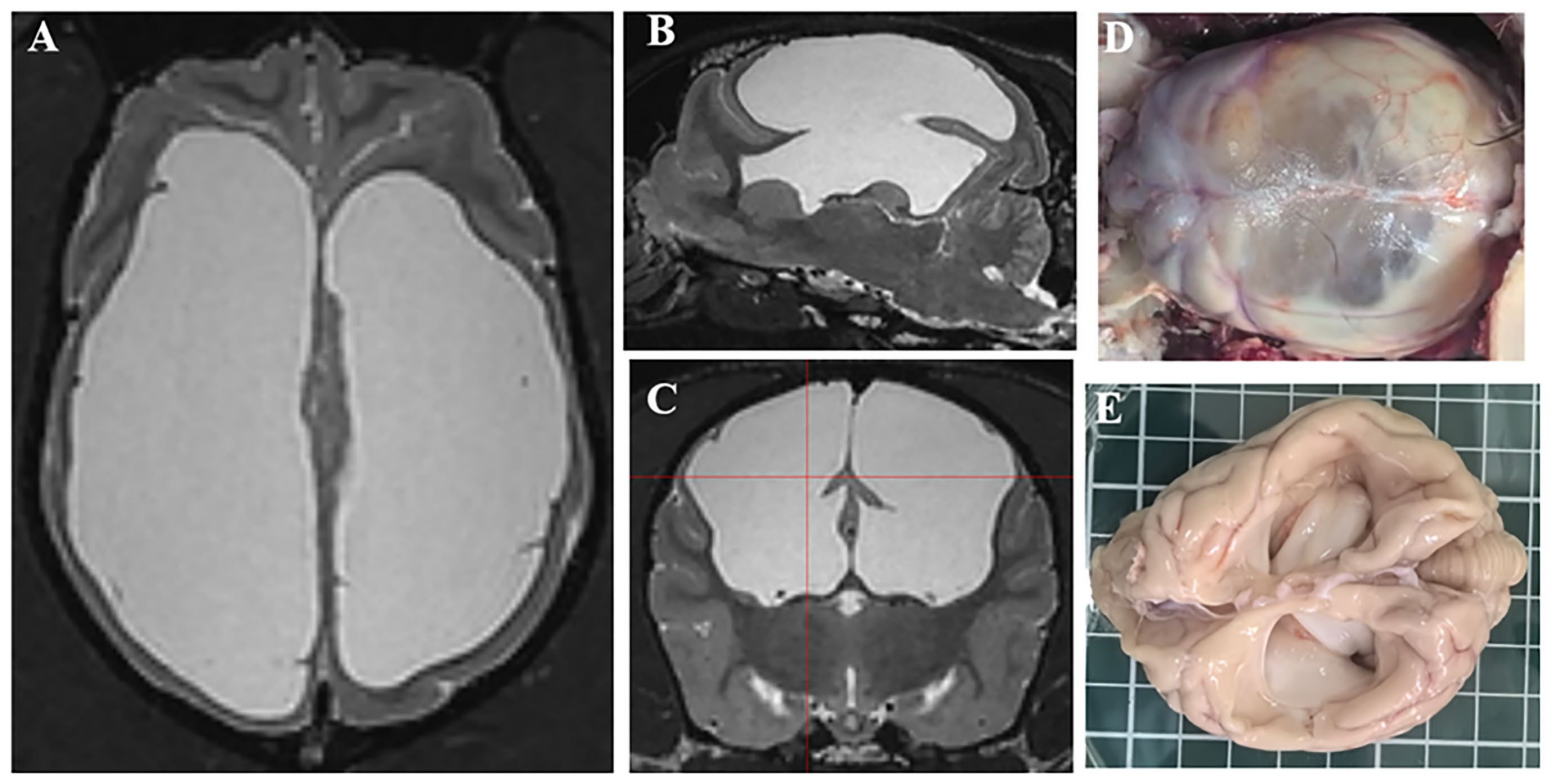
MRI images and photographs of the brain from dog 90044, which exhibited severe hydrocephalus (ventricle-brain index, VBI = 0.72). Dorsal **(A)**, sagittal **(B)**, and transverse **(C)** T2-weighted MRI images showing severe lateral ventricular hydrocephalus. Transverse T2-weighted MR image is located at the middle of the interthalamic adhesion. In this image, the red horizontal line indicates the position of the dorsal image; the red vertical line indicates the position of the sagittal image. Necropsy revealed a submeningeal cavity **(D)** and the dorsal view of the gross brain specimen **(E)** (with no brain structures removed) revealed complete absence of the parietal fiber tracts.

A 1.4-year-old female Beagle (ID: 90044) presented with small body size (9.4 kg), inactivity, and reduced vocalization (Table 1). T2-weighted MR images revealed severe hydrocephalus (Figures 1A–C). Necropsy revealed a submeningeal cavity (Figure 1D) and complete absence of the parietal lobe parenchyma (Figure 1E). Review of its breeding records revealed that among its littermates (*n* = 9), five were stillborn or died preweaning (Table 1). This observation prompted the suspicion of hereditary hydrocephalus within the pedigree. Pedigree tracing to assess heritability revealed that the sire (ID: 11771) exhibited hydrocephalus (VBI = 0.61), while the dam had minimal lateral ventricular enlargement (VBI = 0.52). These findings raise the possibility of hereditary lateral ventricular enlargement within this pedigree, with the proband likely inheriting severe hydrocephalus from the affected sire.

**Table 1:** Distribution of lateral ventricular enlargement based on VBI in control Beagles and 11771 offspring, stratified by age group.

Grade	VBI range	Controls			Offspring sired by 11771			
		All controls *n* = 24 (ratio)	Juveniles *n* = 9 (ratio)	Adults *n* = 15 (ratio)	All cases *n* = 26 (ratio)	Puppies *n* = 16 (ratio)	Juveniles *n* = 5 (ratio)	Adults *n* = 5 (ratio)
Normal	<0.5	7 (29.2%)	5 (55.6%)	2 (13.3%)	9 (34.6%)	9 (56.3%)	0	0
Minimal	0.5–0.55	14 (58.3%)	4 (44.4%)	10 (66.7%)	9 (34.6%)	6 (37.5%)	2 (40%)	1 (20%)
Mild	0.55–0.6	3 (12.5%)	0	3 (20%)	7 (26.9%)	1 (6.3%)	3 (60%)	3 (60%)
Hydrocephalus	≥0.6	0	0	0	1 (3.9%)	0	0	1 (20%)

VBI, ventricle-brain index; Puppy cases: <6 months old; Juvenile cases: 6–12 months old Adult cases: ≥12 months old. n: sample sizes.

### 2.1 Imaging acquisition

A total of 41 offspring were produced from six litters sired by dog 11,771, of which 37 had breeding records and four lacked phenotypic data (Supplementary Table S1). Among the 37 recorded offspring, 4 (10.8%) were stillborn and 7 (18.9%) died prior to weaning. The remaining 26 offspring (comprising 14 females, 12 males) survived to undergo MRI examination (age range 3–20 months, weight range 5.4–12.7 kg, Table 1). To further ascertain whether lateral ventricular enlargement is prevalent in this pedigree, we also performed MRI scans on 24 healthy Beagles (nine juveniles and 15 adults) obtained from another animal center (National Canine Laboratory Animal Seed Center; Supplementary Table S2). All Beagles in this study were classified into three age groups based on established veterinary criteria: puppies (<6 months old), juveniles (6–12 months old), and adults (≥12 months old).

For each MRI session, anesthesia was induced via intramuscular injection of Tiletamine/zolazepam hydrochloride (Zoletil^®^50; Virbac) at 2 mg/kg in combination with dexmedetomidine (Dexdomitor^®^; Orion) at 0.01 mg/kg. Following induction, intravenous propofol (2–3 mg/kg) was administered to enable endotracheal intubation. Anesthesia was subsequently sustained with 1.5% isoflurane under pressure-controlled ventilation (PCV), with the ventilator configured to deliver 12 breaths per minute at an inspiratory pressure of 12 cmH₂O.

T1-weighted and T2-weighted images of all Beagles were acquired on a 3.0 T MR scanner (uMR 790, United Imaging Healthcare, Shanghai, China) equipped with a flexible dog head 24-channel leather coil or 12-channel knee coil at the Core Technology Facility, Kunming Institute of Zoology, Chinese Academy of Sciences. T1-weighted images were obtained using a Fast Spoiled Gradient Echo (GRE-FSP) sequence with the following parameters: in the coronal plane, field of view (FOV) = 120 × 120 mm, matrix = 240 × 240, slice thickness = 0.5 mm, number of slices = 120, averages = 3, repetition time (TR)/echo time (TE) = 11.9/3.8 ms, inversion time (TI) = 900 ms, flip angle (FA) = 8°, and total acquisition time (TA) = 17 min 43 s. T2-weighted images were acquired with a Modulated flip Angle Technique in Refocused Imaging with extended echo train (MATRIX) sequence with the following parameters: in the coronal plane, FOV = 120 × 120 mm, matrix = 240 × 240, slice thickness = 0.5 mm, number of slices = 120, averages = 2, TR/TE = 3,000/405 ms, echo train length (ETL) = 160, and TA = 10 min 12 s.

### 2.2 Grade criteria

The VBI was calculated using dorsal T2-weighted images, defined as the ratio of the greatest continuous distance between the inner margins of the lateral ventricles to the maximum width of the brain parenchyma measured on the same plane (13). The VBI values were measured at three adjacent levels, and the maximum value was used for analysis (Table 1). A four-tier severity grades were assigned according to previously described criteria (13–15): normal (VBI < 0.5), minimal lateral ventricular enlargement (0.5 ≤ VBI < 0.55), mild lateral ventricular enlargement (0.55 ≤ VBI < 0.6), and hydrocephalus (VBI ≥ 0.6).

### 2.3 Major findings

Given the significant disparity in age distribution between the case and control groups (Table 1), we first evaluated the interaction between age and group on VBI. A significant interaction effect was observed (Group × Age interaction, *p* = 1.2 × 10^−3^), indicating that the relationship between age and ventricular size fundamentally differs between the two groups. Therefore, a standard ANCOVA assuming parallel slopes was not appropriate. Instead, we assessed group differences using age-stratified analyses. Within the case group, a Jonckheere–Terpstra test for trend (based on 100,000 permutations) revealed a highly significant increasing trend across the three age strata (puppy, juvenile, adult; JT = 166.5, *p* = 3 × 10^−5^) with the median VBI increased from 0.48 (puppy) to 0.55 (juvenile) and 0.57 (adult). To verify whether the ventricular volume of this Beagle pedigree was abnormally enlarged, we compared the affected dogs with age-matched controls (9 juveniles and 15 adults). Due to the limited sample sizes, non-parametric comparisons were conducted using the Wilcoxon rank-sum test. The analysis revealed significant ventricular enlargement in both juvenile (case median 0.55 vs. control median 0.49; *W* = 44, *p* = 4.8 × 10^−3^) and adult (case median 0.57 vs. control median 0.52; *W* = 68, *p* = 8.3 × 10^−3^) dogs from this pedigree, confirming the presence of abnormal ventricular volume in this pedigree.

When the ventricular size distribution was assessed using a four-tier classification based on VBI (Table 1), the Jonckheere-Terpstra test revealed a significant increasing trend in severity across age groups within the case group from puppy to juvenile and adult (JT = 161.5, *p* = 2.1 × 10^−3^). Specifically, case puppies predominantly presented with normal or minimal ventricular enlargement, juvenile cases shifted entirely toward minimal and mild enlargement, and adult cases progressed to severe phenotypes, including hydrocephalus (Table 1). Age-stratified Wilcoxon rank-sum tests further confirmed the pathological nature of this progression. In the juvenile group, cases demonstrated significantly higher severity than controls (*W* = 41, *p* = 0.010), with a complete absence of normal phenotypes. This disparity persisted into adulthood (*W* = 62.5, *p* = 0.017), where the severe hydrocephalus phenotype occurred exclusively in the case group. In contrast, the control group remained relatively stable across age groups. These findings collectively indicate that the genetic factor in this pedigree leads to age-dependent, progressive ventricular enlargement.

### 2.4 Relationship

Among the six dams, 10012 exhibited hydrocephalus (VBI = 0.69). Both 10012 and 11771 were traced to Qingdao Bolong Beagle Dog Breeding Co., Ltd., and further pedigree investigation confirmed that they share a common great-grandmother, making them second cousins. This familial relationship, together with the presence of ventricular enlargement in both individuals, raises the suspicion of a heritable component underlying the observed ventriculomegaly in this pedigree.

## 3 Discussion

**Figure 2:**
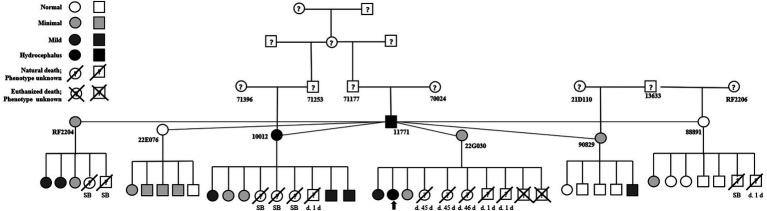
Pedigree of dogs related to dog 11771. Circles represent females, and squares represent males. A question mark indicates dogs with unknown phenotype. A single diagonal line indicates natural death, and crossed diagonal lines indicate euthanasia. The individual pointed to by arrow is dog 90,044.

Hydrocephalus is a common neurologic condition in both dogs (1–3, 15) and humans (22, 23). In this case report, we systematically characterized the ventricular phenotype of a Beagle pedigree (sire 11771, six dams, 26 surviving offspring, Figure 2, Supplementary Table S1). Compared with an age-stratified unrelated control group, both juvenile and adult pedigree offspring exhibited significantly higher VBI and greater severity grades than their age-matched controls. Crucially, within the case group, we observed a highly significant age-dependent increasing trend in both continuous VBI and categorical severity from puppy to juvenile to adult (Jonckheere–Terpstra test, *p* < 0.01). Those findings combined with the presence of hydrocephalus in the sire (VBI = 0.61) and in one dam (10012, VBI = 0.69), who are second cousins (*θ* = 0.03125), raise the suspicion of a heritable component. To our knowledge, this is the first report of a suspected heritable Beagle pedigree with spontaneous, familial ventricular enlargement, providing a unique opportunity to investigate the genetic basis of ventriculomegaly in a breed that typically exhibits a high incidence of asymptomatic distension.

The most striking finding in this pedigree is the clear, age-dependent progressive ventricular enlargement, confirmed by significant trend analyses. When assessed using the four-tier VBI classification, case puppies predominantly presented with normal or minimal ventricular enlargement; however, by the juvenile stage, all cases shifted to minimal or mild enlargement with a complete absence of normal phenotypes. In adulthood, the phenotype further deteriorated, with cases progressing to mild enlargement or severe hydrocephalus, a phenotype observed exclusively in the adult case group. In contrast, the control group remained relatively stable across age groups (Table 1). This finding indicates a clear age-dependent increase in relative ventricular volume. A previous study revealed that the lateral ventricular volume ratio in healthy Beagle-type dogs undergoes a rapid expansion during the initial 60–75 postnatal days, followed by stabilization and sustained constancy until 7 months of age (24). The observed age-dependent elevation in VBI and severity grade within the pedigree suggests a pathologically or genetically mediated phenomenon, as opposed to a physiological developmental trajectory. This progressive trend was also consistent with the observations in the two puppies with a high ventricular ratio (24), suggesting a shared underlying mechanism of delayed-onset or progressive ventriculomegaly.

This study has several limitations. First, the sample size is modest (26 offspring, 24 controls), particularly for the juvenile and adult case comparison (*n* = 5). Second, although significant differences in VBI were observed between age groups within the pedigree (Table 1), and an age-dependent increase in VBI was hypothesized for the puppy cohort, longitudinal evidence to confirm this trajectory is lacking. Third, the absence of genetic marker data precluded formal heritability estimation or linkage analysis. Fourth, post-mortem histopathological assessments were not available for stillbirth or preweaning mortality cases. Finally, as a single-pedigree study, the generalizability of these findings to the broader Beagle population remains uncertain. However, in consideration of animal welfare, we do not plan to continue breeding this pedigree unless there is a need for clinical medicine.

In conclusion, this study presents the first systematic characterization of a suspected hereditary Beagle pedigree with progressive ventriculomegaly. This finding provides an opportunity for early diagnosis, longitudinal monitoring, and development of novel therapeutic approaches for canine hydrocephalus. Beagles allow lifelong longitudinal tracking from early life stages, enabling complete life history documentation, and their lack of owner-pet emotional attachments mitigates confounding biases, making them well-suited for developing new treatment strategies. Investigations into Beagle models not only elucidate mechanisms governing canine health but also hold translational potential to inform precision therapies for human hydrocephalus.

## Data Availability

The raw data supporting the conclusions of this article will be made available by the authors, without undue reservation.
